# Beneficial Properties of Green Tea Catechins

**DOI:** 10.3390/ijms21051744

**Published:** 2020-03-04

**Authors:** Claudia Musial, Alicja Kuban-Jankowska, Magdalena Gorska-Ponikowska

**Affiliations:** 1 Department of Medical Chemistry, Medical University of Gdansk, 80-211 Gdansk, Poland; claudia.musial@gumed.edu.pl (C.M.); alicja.kuban-jankowska@gumed.edu.pl (A.K.-J.); 2 Department of Biophysics, Institute of Biomaterials and Biomolecular Systems, University of Stuttgart, 70569 Stuttgart, Germany; 3 Euro-Mediterranean Institute of Science and Technology, 90139 Palermo, Italy

**Keywords:** green tea, *Camellia sinensis*, catechins, cancer stem cells, anticancer theraphy

## Abstract

Green tea (*Camellia sinesis*) is widely known for its anticancer and anti-inflammatory properties. Among the biologically active compounds contained in *Camellia sinesis*, the main antioxidant agents are catechins. Recent scientific research indicates that the number of hydroxyl groups and the presence of characteristic structural groups have a major impact on the antioxidant activity of catechins. The best source of these compounds is unfermented green tea. Depending on the type and origin of green tea leaves, their antioxidant properties may be uneven. Catechins exhibit the strong property of neutralizing reactive oxygen and nitrogen species. The group of green tea catechin derivatives includes: epicatechin, epigallocatechin, epicatechin gallate and epigallocatechin gallate. The last of these presents the most potent anti-inflammatory and anticancer potential. Notably, green tea catechins are widely described to be efficient in the prevention of lung cancer, breast cancer, esophageal cancer, stomach cancer, liver cancer and prostate cancer. The current review aims to summarize the potential anticancer effects and molecular signaling pathways of major green tea catechins. It needs to be clearly emphasized that green tea as well as green tea catechols cannot replace the standard chemotherapy. Nonetheless, their beneficial effects may support the standard anticancer approach.

## 1. Introduction

*Camellia sinensis* (L.) is one of the oldest and the most popular drinks in the world. Green tea is classified mainly because of the tradition of production of green tea leaf processing, the place of origin as well as by the type of soil on which the bushes have grown. Green tea is grown mainly in Japan, China and Taiwan. The main difference between green tea and black tea is the technological process of their production [1,2,3,4].

There are many types of green tea that are classified according to their taste and antioxidant properties. The most popular type of green tea consumed is Sencha, most often made in Japan [5,6,7]. After proper treatment, the Bancha, Matcha and Gyokuro species are made from Sencha tea. Bancha infusion, compared to Sencha infusion, contains much less caffeine, as well as L-theanine, the amino acid responsible for the formation of proteins responsible for the production of neurotransmitters, insulin and adrenaline [7]. The infusion of Matcha green tea leaves, in contrast to other types of infusions, has the highest amount of caffeine and L-theanine [7]. Available scientific studies indicate that L-theanine significantly modifies the effects of caffeine, reducing its stimulant effect, positively affecting brain work, improving cognitive functions, mood and concentration, and additionally, decreasing blood pressure [6]. Matcha tea has two forms: Matcha–Usucha and Macha–Koicha. Among the Japanese types of green tea infusions, Mecha, Genmaicha, Kukicha, Kamairicha, Kariganech, Konch, Kokeicha, Fukamushicha and Tamaryokucha also stand out. Chinese types of green tea include: Gunpowder, Chun Mee, Lung Ching, Mao Feng, and China Sencha, a Chinese variety of Japanese Sencha infusion [3,4,7].

The technological process has a major impact on the antioxidant potential of green tea. Compared to black tea, green tea has a much higher catechins content. This is a consequence of oxidation of catechins to theaflavins during the fermentation process. In addition, the important fact is that the higher the catechins content in tea, the higher the antioxidant activity. The number of polyphenolic compounds, including catechins, depends on the cultivation conditions of *Camellia sinensis*—climatic as well as agro-technical. It is also noteworthy that as the temperature rises, the antioxidant activity of the green tea infusion increases [6,7]. The percentage content of green tea is not constant, but depends on different environmental factors including growing conditions, soil, climatic conditions, or other external factors such as: light factors, geography, microbes or temperature [4,5,6,7].

The group of catechins (flavan-3-ol) belonging to the group of flavonoids contained in tea include: (−)-epigallocatechin-3-gallate (EGCG), (−)-epicatechin-3-gallate (ECG), (−)-epigallocatechin (EGC) and (−)-epicatechin (EC). Flavonoids are one of the most common and diverse groups of polyphenols. The presence of numerous hydroxyl groups in the molecules gives them strong antioxidant properties [1,2]. The chemical composition of green tea includes more than ten groups of compounds. The main components are phenolic acids, polyphenolic compounds (which include catechins), as well as amino acids, proteins and fats [8,9,10,11,12,13].

Notably, the best source of catechins is unfermented green tea. Depending on the type and origin of green tea leaves, their antioxidant properties may be uneven [13,14,15,16]. Catechins also occur naturally in black tea, coffee, berries, grapes and wine.

Due to the numerous health-promoting properties of catechins, it is recommended to include particularly products containing catechins in the daily diet [17]. Anti-inflammatory and antioxidant as well as chemopreventive activity are considered as the most important action of the catechin group [13,14,15,16,17,18].

The basic functions of catechins include their antioxidant effects: scavenging of reactive oxygen species, inhibition of the formation of free radicals and lipid peroxidation. Available literature data indicate that the antioxidant activity of catechins contained in green tea and their significant impact on the prevention of civilization diseases are largely dependent on the presence of structural groups in the molecules, as well as the number of hydroxyl groups [3,4]. Green tea may exert the prevention effect in various types of cancer including lung, esophagus, stomach, intestinal, pancreatic, breast, prostate or bladder cancers [14,15,16,17].

However, it is worth taking into consideration the oxidative potential of catechins, for example, when using green tea in the form of dietary supplements, as a result of which there is a possibility for the formation of very highly reactive metabolites with quinone structure. Quinones, as a result of redox reactions, have also the potential to generate high amounts of reactive oxygen species [19].

## 2. Health-Promoting Properties of Green Tea

As mentioned above, green tea is not produced as a result of fermentation, unlike the production of black tea, during which fermentation to Oolong takes place (which is partially fermented). The process of full fermentation into black tea is a result of the influence of enzymes on catechin polyphenols found in the leaves of the tea bush [14]. The technology of processing *Camellia sinensis* leaves makes each type of tea have a different effect and has other biologically active ingredients and health-promoting properties [14,15].

The health-promoting properties of green tea are due to the presence of polyphenols, in particular, flavonols and flavanols. Clinical studies, in vivo and in vitro experiments, confirm their antioxidant and anti-inflammatory effects. Catechins are the dominant polyphenols in green tea, whose antioxidant activities result from the neutralization of free nitrogen and oxygen radicals, as well as the ability to chelate metal ions in redox reactions. Numerous scientific studies indicate the antitumor effects of polyphenols contained in green tea leaves due to inhibition of cell division as well as the induction of phase II antioxidant enzymes, e.g., superoxide dismutase, glutathione-*S*-transferase as well as glutathione peroxidase and reductase. The described result concerns the research on the effects of polyphenols on oxidative stress in vivo. The study showed that consumption of green tea within 4 months in an amount of four glasses per day reduced urinary levels of 8-hydroxydeoxyguanosine. The effects of green tea polyphenols on inhibition of the growth of cancer cells and reduction of the risk of cancer are confirmed by numerous scientific studies in the field of prostate, pancreatic, breast and stomach cancers [14,15,16]. It is worth emphasizing that green tea may support chemotherapeutic as well as preventive effects, however, it cannot replace pharmacological treatment. Notably, polyphenols, including catechins, are able to induce cancer cell death while not affecting healthy cells [9,14,17].

## 3. The Chemical Composition of Green Tea

From a chemical point of view, green tea has a protein content of about 15–20%, which include amino acids such as l-theanine [10], tyrosine, tryptophan, threonine, 5-*N*-ethylglutamine, glutamic acid, serine, glycine, valine, leucine, aspartic acid, lysine and arginine. It also contains trace elements such as magnesium, chromium, manganese, calcium, copper, zinc, iron, selenium, sodium cobalt or nickel, and carbohydrates such as glucose, cellulose and sucrose [9,10,11,12,13,14,15,16,17,18,20,21]. In addition, green tea is rich in sterols and lipids—linoleic and α-linolenic acid, and vitamins B2, B3, C—of which the most is in Gyokuro tea (about 10 mg) and Sencha (4 mg), vitamin E and trace amounts of vitamin K. Vitamin A only occurs in Matcha tea. It is also important that green tea is extremely rich in macroelements; it is a source of fluorine, iodine as well as phosphorus. The diphenylpropanoid skeleton (C_6_C_3_C_6_) content is also typical feature of green tea [10,11,12]. In addition, green tea is rich in xanthine bases, which include theophylline and caffeine [20], as well as pigments such as carotenoids and chlorophyll. It is worth noting that the chemical composition of green tea also includes phenolic acids, which include gallic acid and volatile compounds including alcohols, esters, hydrocarbons and aldehydes. Phenolic acids, which include proanthocyanidins, as well as gallic acid esters with monosaccharides, have a huge impact on the qualities of green tea infusion.

**Figure 1:**
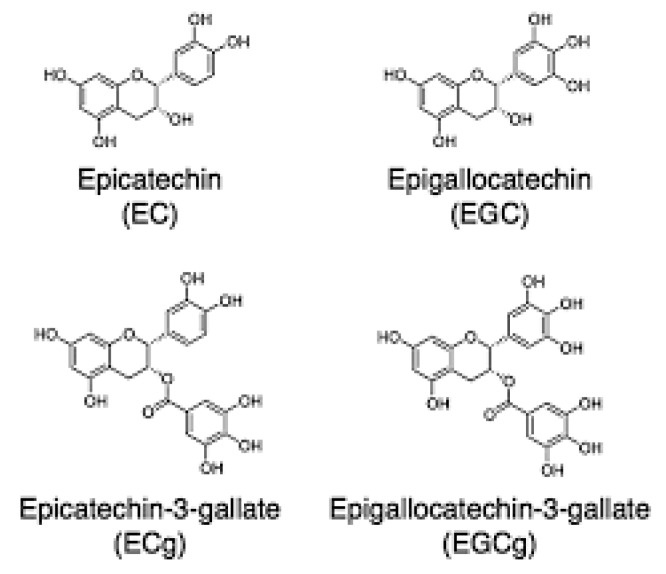
This figure presents chemical structure of green tea catechins.

The phenolic acids mentioned earlier belong to the group of polyphenols, together with flavonoids, flavandiols and flavols. Available data indicate that these compounds can constitute up to 30% of the dry matter of green tea. Catechins are the standard green tea flavonoids. Green tea contains a much higher amount of catechins than black tea or Oolong. As mentioned above, the group of catechins include EGCG, ECG, EGC and EC [10,11,12,13]. Below (Figure 1) are the chemical structure of green tea catechins. In addition to the number of hydroxyl groups, their distribution is equally important, taking into account the antioxidant activity of catechins [16]. Catechins, with a catechol group have lower antioxidant potential compared to catechins with a pyrogalol group. However, the antioxidant efficacy of catechins depends not only on the chemical structure, but also on the environmental conditions [10,11,12,13,14,15,16,17,18].

The individual chemical components of green tea have a fundamentally different effect on particular types of cancer. Available data indicate that ascorbic acid, arginine, proline, lysine and EGCG were reported to have a positive effect on tumor growth reduction [12,13,14,15,16,17].

**Figure 2:**
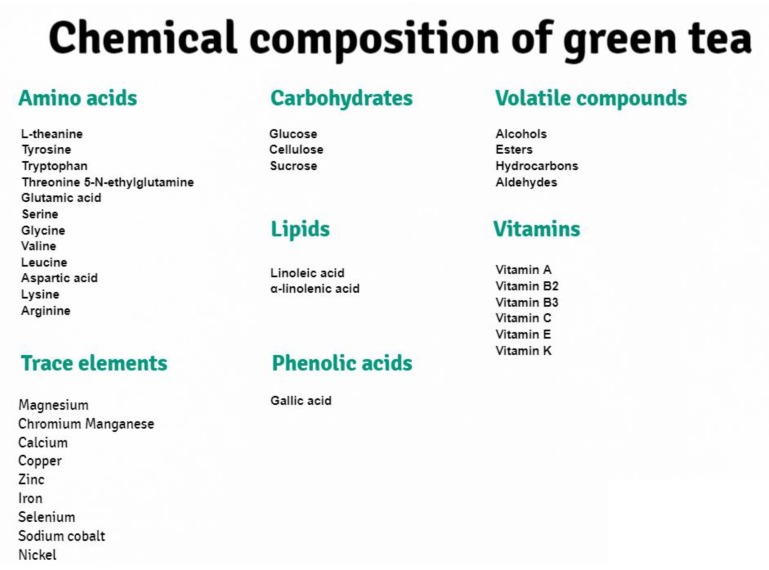
Chemical compounds of green tea.

On figure below (Figure 2), we present the chemical composition of green tea, broken down into lipids, amino acids, trace elements, phenolic acids, vitamins, carbohydrates and volatile compounds.

## 4. Catechins: Modes of Action

EGCG is so far the best studied catechin derivative. The amount of catechins in green tea depends primarily on its variety, the method of its cultivation and leaf processing, as well as the brewing time and temperature. Studies show that catechins achieve the highest stability in the range of pH between 4 and 6 [9,10,11,12,13].

Available data indicate antitumor [9], antioxidant [17], anti-inflammatory [22], anti-microbial, anti-viral [23,24,25], anti-diabetic, anti-obesity and hypotensive effects [23,24,25] of catechins. Their beneficial effects on Gram-positive and Gram-negative bacteria, viruses, fungi and prions also need to be emphasized [1].

Catechins also act as the metal ion chelators for copper ions and iron ions. The specific chemical structure of polyphenols (the presence of a minimum of five hydroxyl groups) contained in green tea has a significant impact on the antioxidant capacity [26,27,28,29]. Chelation of transition metal ions is possible due to the di/tri-hydroxy structure of the B and D rings [26], as well as the meta-5,7-dihydroxy group at the A ring [1,30,31]. However, under specific conditions, they may have pro-oxidative effects [32]. The control of catechins under intracellular pool of nitro-oxidative stress is mainly responsible for their anticancer properties [33]. Therefore, polyphenolic compounds that bring health-promoting properties for the body can also result in the opposite effects if very high doses of catechins are used [19]. The result is induction of pro-oxidative stress, as well as oxygen damage to cellular components. In addition, polyphenols also have a pro-oxidative effect in the presence of tyrosinase or peroxidase, i.e., oxidizing enzymes. In addition, the pro-oxidative effect is closely related to inflammatory processes [19]. Analogously to the antioxidant properties, the pro-oxidizing properties of catechins depend identically on such factors as the number of hydroxyl groups in the molecule. During the process of polyphenol oxidation, cellular molecules are damaged by reactive oxygen species as well as electrophilic quinones. This factor is crucial in the etiopathogenesis of degenerative diseases and a carcinogenic process [19].

Catechins, as well as other active ingredients derived from green tea, can also repair DNA damage caused by UVB radiation. Available data point to the high effectiveness of green tea active ingredients in order to prevent ultraviolet radiation damage to the skin [20].

## 5. Anticancer Potential of Green Tea Catechins Based on In Vitro and In Vivo Studies

The most potent bioactive ingredient in green tea is EGCG, containing eight hydroxyl groups, named as the main green tea polyphenol [20,34,35]. The cell-death-inducing effect of catechins has been previously confirmed in prostate cancer animal model [34]. Many studies have been conducted to confirm the induction of apoptosis and cell cycle arrest by EGCG, e.g., in colon cancer HCT-116 cells [36,37]. It is believed that the main antitumor mechanism of EGCG is the inhibition of metalloproteinase activity. This hypothesis has been supported by study indicating the reduction of prostate cancer metastases after oral supplementation of green tea catechins [33]. Green tea catechols were also proven to inhibit lung melanoma metastasis in animal model [35]. Moreover, a positive relationship between green tea intake and the development of bladder cancer was also reported. There are also studies confirming the preventive function of colorectal adenoma after consuming ten cups of green tea of 150 mL each [35].

Breast cancer is one of the most common cancers around the world among the female population, breast cancer cells have been repeatedly subjected to scientific and clinical research to determine the effect of green tea catechin derivatives, including chemo-preventive as well as synergistic effects along with chemotherapy [38]. It is well known that the main catechins of green tea may induce anti-angiogenic and anti-proliferative effects in cancer cells, which results in their potential chemo-preventive properties. The effectiveness of green tea catechins in patients with breast cancer has been evaluated in a clinical trial, using Polyphenon E as a supplementation, consisting of succeeding catechins: EC, EGC, ECG, and the main EGCG [38]. Each capsule contained a decaf EGCG mixture with 200 mg content. During the I phase of the clinical trial, a limit of 1200 mg EGCG was established as acceptable for future safety. The study was conducted on a group of patients with breast cancer lacking the hormone receptor [38]. 

Lung cancer is currently the most common malignancy in the world. The effect of oral supplementation of EGCG on H1299 human non-small cell lung cancer xenograft in case of animal (mice) models have been evidenced. Research results indicate an increase in apoptosis in cancer cell death as well as inhibition of tumor growth in lung cancer [39]. In addition, oral supplementation of EGCG induced the formation of reactive oxygen species in the mitochondria of lung cancer cells, possibly due to the limited number of antioxidant enzymes in these cells [39]. It was proven that catechins derived from green tea, while added to the medium used in cell culture, increase level of oxidative stress, leading to apoptosis [34].

Therefore, many in vivo studies determine the effect of the amount of consuming green tea on the reduction of incidence of malignant tumors, including colorectal cancer, stomach cancer, liver cancer [19] or lung cancer [38]. These results concern drinking more than ten cups of green tea infusions per day [40]. However, on the other side, one study indicates an increased risk of developing bladder cancer when consuming five to nine cups of green tea infusions per day. Another interesting result of the study carried out by the Taniguchi group is the oral consumption of the main catechins of green tea, EGCG, and its positive anticancer effect established in melanoma animal model [41].

The anticancer potential of EGCG has also been studied in cancer stem cells [33]. Stem cells, or precursor cells, are characterized by the ability to proliferate, i.e., self-renew, and maintain a constant, unchanging number of cells; they also have the ability to differentiate into an appropriate cell type. Notably, green tea extract and EGCG inhibit cell growth in these cellular and animal models [42,43]. In vivo and in vitro studies report that cancer stem cells are responsible for cancer renewal as well as metastasis [33]. Available data indicate that tumor stem cells overcome the epithelial–mesenchymal transition during the metastasis process [33]. This process allows the cancer cells to move towards the blood vessels. It is noteworthy that cancer stem cells, when compared to a cancer cell, show a much greater capacity for oncogenesis [33]. After analyzing the available scientific research on the use of green tea catechins on cancer stem cells, we found information indicating the effect of Matcha green tea catechins on the oxidative phosphorylation of MCF-7 breast cancer stem cells [33]. In addition, treatment with Matcha green tea extract of MCF-7 breast cancer cells also affects the regulation of the cell cycle, and causes a significant effect on the IL-8 pathway involved in the proliferation and angiogenesis of migratory cancer cells [33].

Molecular Signaling Pathways in Anticancer Effects of Green Tea Catechins

Cell signaling pathways, responsible for maintaining a homeostasis between cell proliferation and death, have emerged as rational targets for anticancer strategies.

As mentioned above, green tea catechins, especially the most potent EGCG, induce apoptosis in different cancer models. Notably, it is able to induce both intrinsic (mitochondrial) and extrinsic (death receptor) apoptotic pathways [44]. Nuclear condensation, caspase-3 activation, and poly(ADP)ribose polymerase cleavage are the main apoptotic features observed after treatment with green tea catechols [45]. In addition, the anticancer mechanism of EGCG also includes activation of BAX, depolarization of mitochondrial membranes, and cytochrome c release into cytosol [46].

The induction of cell cycle arrest and apoptosis are the main strategies of regulating cell proliferation. Indeed, green tea catechols regulate both the G1/S and G2/M transition and inhibit an increase in the number of cells and DNA synthesis [44]. Importantly, EGCG induces apoptosis and cell cycle arrest in many cancer cells without affecting normal cells [12]. EGCG directly inhibits the cyclin-dependent kinases which is the primary event in cell cycle progression [44]. EGCG also induces the expression of p21 and p27 while decreasing the expression of cyclin D1 and the phosphorylation of retinoblastoma [44].

The molecular signaling pathways regulated by green tea catechols resulting in their pro-apoptotic and anti-proliferative effects include, among others, inhibition of nuclear factor-κB (NF-κB) which is the crucial oxidative stress-sensitive transcription factor [14,35] NF-κB plays a critical role in the regulation of a variety of genes important in cellular responses, including inflammation, proliferation and cancer cell death. In addition, catechins contained in green tea, and above all the main catechin EGCG, activate endothelial nitric oxide synthase (eNOS) [42,47].

Inhibition of the mitogen-activated protein kinases (MAPKs), ERK, JNK, and p38 is implicated in many patho-physiological processes, such as cell proliferation, differentiation, and cancer cell death [39,46]. In addition, EGCG is known to lead to the induction of apoptosis in cancer cells by inhibiting tumor necrosis factor α activity (TNF-α) [42].

Another molecular signaling pathway event regulated by green tea catechols is the inhibition of the epidermal growth factor receptor (EGFR)-mediated signal transduction pathway. EGFR is a plasma membrane glycoprotein with an extracellular ligand-binding domain, a single transmembrane region, and an intracellular domain that exhibits intrinsic tyrosine kinase activity. Overexpression of EGFR produces a neoplastic phenotype in tumor cells. Notably, EGCG inhibits the activation of the EGFR, HER2, and multiple downstream signaling pathways in colon cancer cell lines [12,35].

Notably, molecular signaling pathways of green tea catechols involve the additional inhibition of insulin-like growth factor-I (IGF I)-mediated signal transduction pathway [12]. As evidenced, green tea catechins significantly reduce IGF-I protein levels in prostate cancer animal models [48].

**Figure 3:**
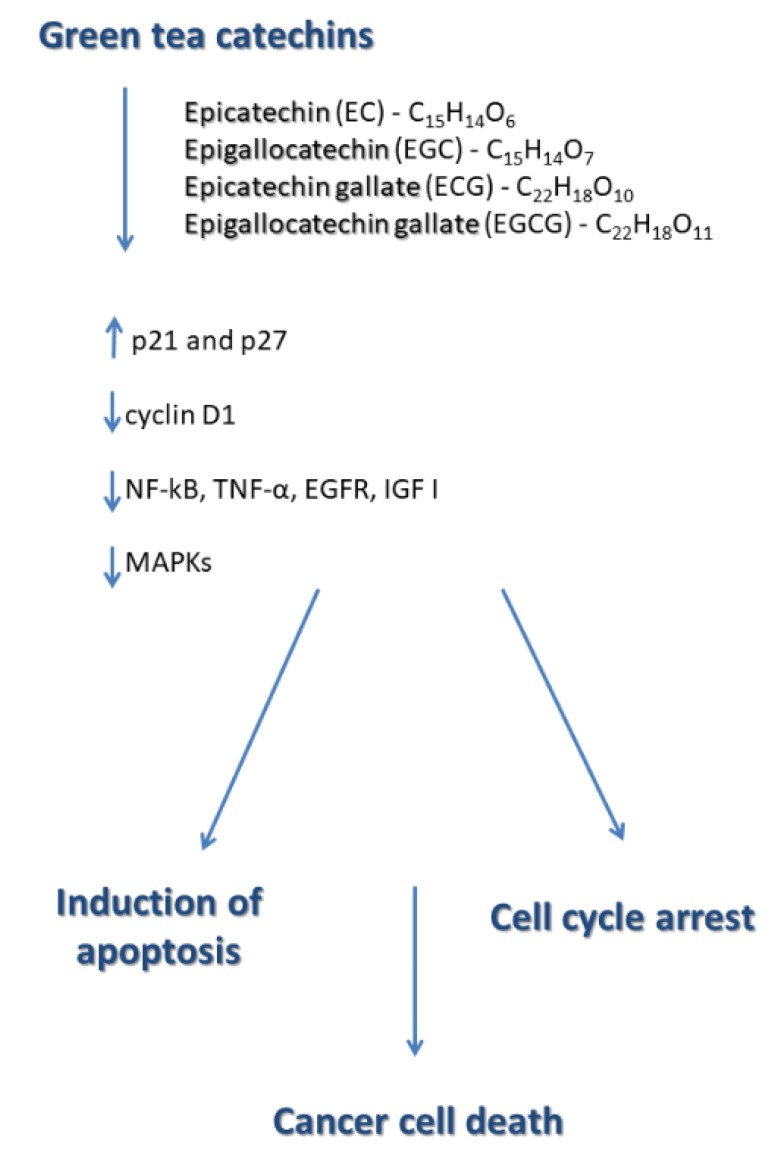
Summary of molecular signaling pathways of green tea catechin.

Available literature data indicate that polyphenols derived from green tea exert their antitumor activity due to modification of histones, micro-RNA as well as DNA methylation [40]. Figure 3 presents a summary of molecular signaling pathways of green tea catechin.

## 6. Differences between Black Tea and Green Tea

Black tea significantly differs from green tea, primarily in terms of chemical composition as well as the fermentation and oxidation process. Black tea, like green tea, is rich in a number of catechins as well as theaflavin; namely, Theaflavin (TF1), Theaflavin-3-monogallate (TF2a), Theaflavin-3′-monogallate (TF2b), and Theaflavin-3,3′-digallate (TF3). Numerous articles describe the molecular mechanism of black tea extraction, as well as individual theaflavins. According to the data, black tea in addition to catechins and theaflavins consists of phenolic acids, flavanols, tearubigins, amino acids, proteins, methylxanthine, and mineral compounds and volatile substances [49]. However, it is known that both tearubingins and theaflavins are products of the tea polyphenols.

The main molecular mechanisms of black tea polyphenols include the activation of mitochondrial cell death signaling pathways and reactive oxygen species-scavenging effects. The molecular effects of polyphenols contained in black tea additionally include activation of nuclear factor erythroid 2-related factor 2 (Nrf2), which is responsible for controlling gene expression as well as regulating antioxidant and detoxifying enzymes [12,14,35,48,49,50]. Notably, the anti-estrogenic impact of black tea consumption may significantly reduce the risk of malignant neoplasms in women [51].

**Figure 4:**
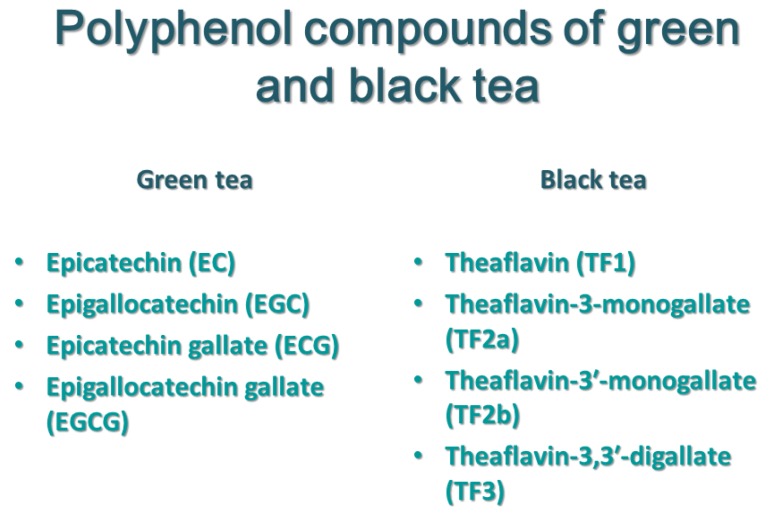
Comparison of polyphenol content in green and black tea.

Theaflavin 1 was found to prevent lung tumorigenesis via induction of apoptosis, down-regulation of fatty acid synthase and COX-2 in cellular and animal models. Theaflavin 2 induced cell death by regulating BAX and p53 protein in the HeLa and WI38VA cervical cancer cell line [45,52,53]. On figure below (Figure 4), we present a comparison of polyphenol content in green and black tea, broken down into theaflavins and catechins.

## 7. Conclusions and Future Perspectives

A number of reports suggesting the beneficial effect of green tea polyphenols on cancer prognosis and prevention as an adjunct to pharmacological treatment have been reported [6,9].

There is a lot of in vivo evidence confirming that consumption of green tea in the form of a drink or dietary supplement exerts the anticancer properties [43,47,54]. The other valuable properties of green tea catechols involve their anti-viral, anti-bacterial, anti-aging, and hypotensive effects [4,5,9,12,27]. Nonetheless, it needs to be clearly emphasized that green tea as well as green tea catechins cannot replace standard chemotherapy. However, their beneficial effects may support anticancer effects and can be used as an adjunct [43,47,54,55].

All in all, polyphenols, and especially the main catechin of green tea, EGCG, brings promising results in the prevention of breast, lung, prostate, stomach, and pancreatic cancers.
